# Pellagra

**Published:** 1915-08

**Authors:** R. H. L. Bibb


### Pellagra.

BY DR. R. H. L. BIBB.

It may be written of pellagra, as has been written of beri-beri, that it is "a collective name for a symptom complex which is produced under different circumstances by different causes."

Beri-beri, like pellagra, is one of the "nutritional diseases" due to nitrogen or phosphorus starvation, or both, and the fact that beri-beri occurs where "polished rice" is, rather was, then unknown, is proof positive that some other "neuritis preventing principle" than the pericarp was absent in the food or drink of those thus afflicted, or that some other agent or influence played the part of *casus morbi* in the disease or assisted in its production.

Pellagra, in many respects, very closely resembles beri-beri, and, as with its congener, nearly everything has been accused of producing it. Formerly, the zeists thought it due alone to spoiled corn, or to fungi found associated with imperfectly ripened or damp, musty, moldy corn; but the fact that pellagra occurs as often in those who eat no corn food as it does in those who live chiefly upon it, compels us to deny that any sort of corn eating is the sole cause of pellagra. The same argument constrains a denial that pellagra is caused by consumption of the so-called rapidly drying oils, such as cotton seed and sunflower seed, oils, etc. The *simulium*—the buffalo gnat—has existed in Texas for years, but it is only within recent times that pellagra appeared among us. If that insect is the cause of the disease it is pertinent to inquire why have we not had it with us from the first settlers, as we have had malarial fevers.

The latest "Richmond in the field," the latest theory concerning the causes of pellagra with which the writer is acquainted, is the "mineral acidosis" theory promulgated and urged by Ceucelli, Scala and Alessandrini. An abstract of this theory, from the *London Lancet* of April 17, 1915, appears in the *Journal of the A. M. A.*, May 15, 1915. According to this theory, pellagra is due to poisoning brought about by silica in colloidal solution in waters used for domestic purposes. Rain water, falling upon the earth, percolates the deep and superficial layers of clay, which is silicate of alumina, causing a hydrolysis which produces, in turn, silicic acid and hydrate of alumina, which pass into the water as colloids. From the chemical action that follows, the alumina is precipitated, in part, in the form of colloidal silica-alumina, the remainder continues in suspension as a very finely divided colloid which, in due time, bring about the "mineral acidosis," the anatomic, pathologic and metabolic alterations, and the syndrome of symptoms to which the name of pellagra is applied.

Basing their therapy upon these considerations, Scala and Alessandrini concluded that the acidosis would yield to alkaline injections, such as trisodium citrate. They also considered that the pellagra inducing water could be corrected and freed from the injurious substances by placing small pieces of lime in the reservoirs and other receptacles for holding water.

Applying their theory to cases of induced pellagra in animals, and to human beings who had suffered from the disease for longer or shorter periods of time, it was found in the highest degree satisfactory from every standpoint. "Patients who had been ill for a long time improved and were cured in a relatively short space of time, without any change having been made in their mode of life, surroundings or diet."

Assuming the chemical changes to occur as stated above, and that the effects following Scala's and Alessandrini's treatment of pellagra are true, it follows that "mineral acidosis" is as completely proven to be the cause of pellagra as is the consumption of polished rice to be the cause of beri-beri. The theory is an attractive one, but will not explain the occurrence of pellagra among those who use cistern water, exclusively, for drinking and cooking purposes unless it can be shown that colloidal silica-alumina exists in rain water at the time it reaches the cistern, or is formed therein afterwards—in metallic, wood and subterranean cisterns as well.

This subject will be discussed more fully at some subsequent date; in the meantime future revelations will be anxiously looked for. The alarming increase in the number of pellagrins; the want of unity of opinion as to the cause of pellagra; *the utter absence of a demonstrated specific cause of the disease*—that will explain every case, as in other specific affections—the various methods of treatment all claiming satisfactory results, and the contradictory results in the experience of others with similar methods of treatment, and with no treatment, save improved hygiene and alimentation, invests the disease with more than ordinary interest.

---

"Look here!" said an excited man to a druggist. "You gave me morphine for quinine this morning."

"Is that so?" replied the druggist. "Then you owe me twenty-five cents."—*Christian Register.*

---

It was a very small pupil who astonished his father, a practioner of medicine, by propounding the following question:

"Papa, do you know what the great Napoleon's nickname was?"

Wishing that his son might have the pleasure of bestowing this information, his father evaded a reply by asking another question: "What was it, son?"

His state of mind can be imagined when the little fellow proudly responded:

"He was known as the Little Corpuscle."—*Youth's Companion.*

